# Postoperative Systemic Immune-Inflammation Index (SII): A Superior Prognostic Factor of Endometrial Cancer

**DOI:** 10.3389/fsurg.2021.704235

**Published:** 2021-10-22

**Authors:** Yihong Huang, Yu Chen, Yan Zhu, Qing Wu, Chengyun Yao, Hongping Xia, Congzhu Li

**Affiliations:** ^1^Department of Gynecologic Oncology, Cancer Hospital of Shantou University Medical College, Shantou, China; ^2^Department of Gynecology and Obstetrics, Wuxi Maternal and Child Health Hospital Affiliated Nanjing Medical University, Wuxi, China; ^3^Jiangsu Cancer Hospital, The Affiliated Cancer Hospital of Nanjing Medical University, Jiangsu Institute of Cancer Research, Nanjing, China; ^4^State Key Laboratory of Reproductive Medicine, Key Laboratory of Antibody Technique of National Health Commission, School of Basic Medical Sciences, Sir Run Run Hospital, Nanjing Medical University, Nanjing, China

**Keywords:** endometrial cancer, postoperative systemic immune-inflammation index, prognosis, systemic inflammatory response, nomogram

## Abstract

**Objective:** This study evaluates the preoperative and postoperative systemic immune-inflammation index (SII) capacity to predict the prognosis of patients with endometrial carcinoma after the operation and build a nomogram model to assist clinical practice.

**Methods:** The retrospective study included 362 consecutive patients with surgically resected endometrial cancer between January 2010 and June 2015 at The Affiliated Cancer Hospital of Shantou University Medical College. Blood routine was examined within 1 week before surgery to calculate SII, NLR, PLR, and MLR and 3 days after surgery to measure SII. The Pearson's χ^2^-test or Fisher's exact test was used to explore their relationship to clinical variables. The univariate and multivariate survival analyses were performed by Cox regression to identify the independent prognostic indicators. The Kaplan–Meier method with the log-rank test was used to generate the overall survival (OS) curves. R software was used to generate the receiver operating characteristic (ROC) curve and then it got the optimum cutoff value through the maximum Youden index. A nomogram model was formed with systemic immune inflammation and clinical factors.

**Results:** The preoperative SII was related to age (*p* = 0.009), FIGO stage (*p* = 0.02) and menopause (*p* = 0.014). The postoperative SII was associated with menopause (*p* = 0.014). Univariate analysis indicated that FIGO stage, lymphatic invasion, depth of myometrial invasion, postoperative chemotherapy, postoperative radiotherapy, preoperative SII, NLR, PLR, MLR, CRP, CA125, and postoperative SII were predictors of OS (*p* < 0.05). Multivariate analysis showed that lymphatic invasion and postoperative SII were independent prognostic factors of OS (*p* < 0.05). The nomogram model was visualized precisely to reflect the prognosis with a C-index value of 0.866 in this model.

**Conclusion:** The postoperative SII is the independent prognostic factor in patients with endometrial carcinoma after the operation and contributes to poor outcomes. However, after surgery, the preoperative SII and preoperative NLR, PLR, and MLR are not associated with OS endometrial carcinoma. Making good use of the nomogram model would contribute to better subsequent therapy.

## Introduction

The continued increase in incidence for endometrial cancer (1.3% per year from 2007 to 2016) and endometrial cancer survival has not improve due to delayed marriage and childbearing and the rising rate of obesity (1). Endometrial cancer subtypes are classified into type I and II cancers. Type I cancers are estrogen-dependent, connected with abnormal uterine bleeding, diabetes, obesity, hyperestrogenism, hypertension, delay of menopause, and functional ovarian cancer. The most common pathology is endometrioid carcinoma. By contrast, type II cancers are estrogen-independent, have less differentiation, and have higher malignancy. The pathological types are rare, such as serous carcinoma, clear cell carcinoma, and carcinosarcoma. The typing of dualism exists in conformity between part case and pathological features (2). The Cancer Genome Atlas (TCGA) (3) based on molecular characteristics classified into four subtypes, including POLE-mutation (POLE mt), microsatellite instability (MSI), low-copy-number, and high-copy-number (4, 5). Nevertheless, these novelty subtypes are not yet widely used in clinical practice because of the high cost and technical problems.

Most endometrial carcinoma in the early stage undergoing surgical removal has a better outcome. However, existing research indicates that the primary reason for the increased mortality rate of endometrial cancer is recurrence after surgery (6, 7). Although the traditional clinical features, such as FIGO stage, tumor grade, histological type, node metastasis, and myometrial invasion, are currently known as risk factors (8), they cannot accurately predict the prognosis of endometrial cancer. It is important to recognize patients diagnosed with early stage tumors and high-risk factors and provide remedial measurement in a timely manner. Therefore, the core of the clinical study is to find the optimal predictive prognostic factors of endometrial cancer. Previous research proves that systematic immune inflammation plays a part in the mechanism of tumor initiation, progression, and metastasis. Systematic inflammation response biomarkers such as neutrophil-lymphocyte ratio (NLR), platelet-lymphocyte ratio (PLR), monocyte-lymphocyte ratio (MLR), and systemic immune inflammation (SII) (9) before surgery were highly associated with several kinds of cancers, such as esophageal squamous cell carcinoma (10), pancreatic cancer (11), hepatocellular carcinoma (12), bladder cancer (13), and endometrial cancer (14), cervical cancer (15), predicting poor prognosis. The postoperative NLR could also reflect the influence of operation and the systemic inflammation response (16, 17). The majority of previous research has concentrated on the value of preoperative NLR and PLR as predictors in endometrial carcinoma. However, less research focuses on the relationship between SII and endometrial carcinoma.

Our research aims to evaluate the predicted prognosis of preoperative and postoperative SII in patients with endometrial cancer. Meanwhile, based on the systemic immune-inflammation index and clinical characteristics, a nomogram model was formed to visualized the prognosis of endometrial cancer following the operation.

## Materials and Methods

### Patients

We retrospected clinicopathologic data of 362 patients diagnosed with endometrial cancer surgically resected between January 2010 and June 2015 at The Affiliated Cancer Hospital of Shantou University Medical College. The inclusion criteria were patients diagnosed with endometrial carcinoma and who received surgery with hysterectomy bilateral salpingo-ovariectomy and/or lymph node dissection. The exclusion criteria included (1) patients who underwent chemotherapy and/or radiotherapy before surgery, (2) patients who had an acute or chronic infection, (3) patients who had blood disease or autoimmune disease, (4) patients lost to follow-up or with incomplete data, (6) patients who failed with a standard surgical approach, (7) patients diagnosed with other malignant tumors.

### Clinicopathological Parameters

Clinical data was received from the electronic medical record management system of the hospital, including age at the time of operation, menopausal status, body mass index (BMI), complications, FIGO stage, tumor grade, pathological type, depth of myometrial invasion (MI), lymphatic vasculature space infiltration (LVSI),levels of serum CA125, operation ways, postoperative adjuvant therapy (chemotherapy or radiotherapy).

The surgery approach of laparoscopy was first applied in the department of gynecologic oncology at the Cancer Hospital of Shantou University Medical College in the year of 2014. Tumor grade was based on the WHO histological classification criterion. The primary endpoint was overall survival (OS). The follow-up time was calculated from the date of the surgery to death or the last date of follow-up (June 30, 2020). The follow-up was regular, every 3 months for the first 3 years after discharge from the hospital and, after that, every 6 months until once a year 5 years later. The follow-up content included routine gynecological checks, serum tumor markers, such as CA125, pelvic ultrasonography, computed tomography (CT) scans, and/or magnetic resonance imaging (MRI).

### Calculation Formula of SII, NLR, PLR, and MLR

Peripheral blood was acquired within 1 week before surgery and 3 days following the operation, including preoperative and postoperative platelet counts, neutrophil counts, lymphocyte counts, and monocyte counts. The complete blood counts are from previous electronic medical records. The systemic immune-inflammation index calculation formula is as follows: platelet counts multiplied by neutrophil counts and then divided by lymphocyte counts SII, neutrophil count divided by lymphocyte counts equals NLR, platelet counts divided by lymphocyte counts equals PLR, monocyte counts divided by lymphocyte counts equals to MLR.

### Statistical Analysis

SPSS version 19.0 (IBM Corp., Armonk, NY, USA) was used in our study to conduct statistical analyses. The Pearson chi-square and Fisher's exact tests were used to explore the relationship between preoperative SII NLR, PLR, MLR, and postoperative SII and clinical variables. The univariate and multivariate stepwise survival analyses were performed by Cox regression analysis to identify the independent prognostic indicators. R 4.0.3 software (http://www.Rproject.org) was used to generate the receiver operating characteristic (ROC) curve of preoperative SII NLR, PLR, MLR, and postoperative SII and then the value of the optimum cutoff value through the maximum Youden index. The preoperative SII NLR, PLR, MLR, CA125, CRP, and postoperative SII were divided into a high and low group according to the optimum cutoff value. Nomogram for OS based on postoperative SII and other clinicopathological factors was established using R 4.0.3 software (http://www.Rproject.org). Concordance index (C-index) and calibration curve were used to reflect the discriminative ability of the nomogram model. A *P* < 0.05 was considered statistically different in our study.

## Results

### Patient Characteristics

**Figure 1:**
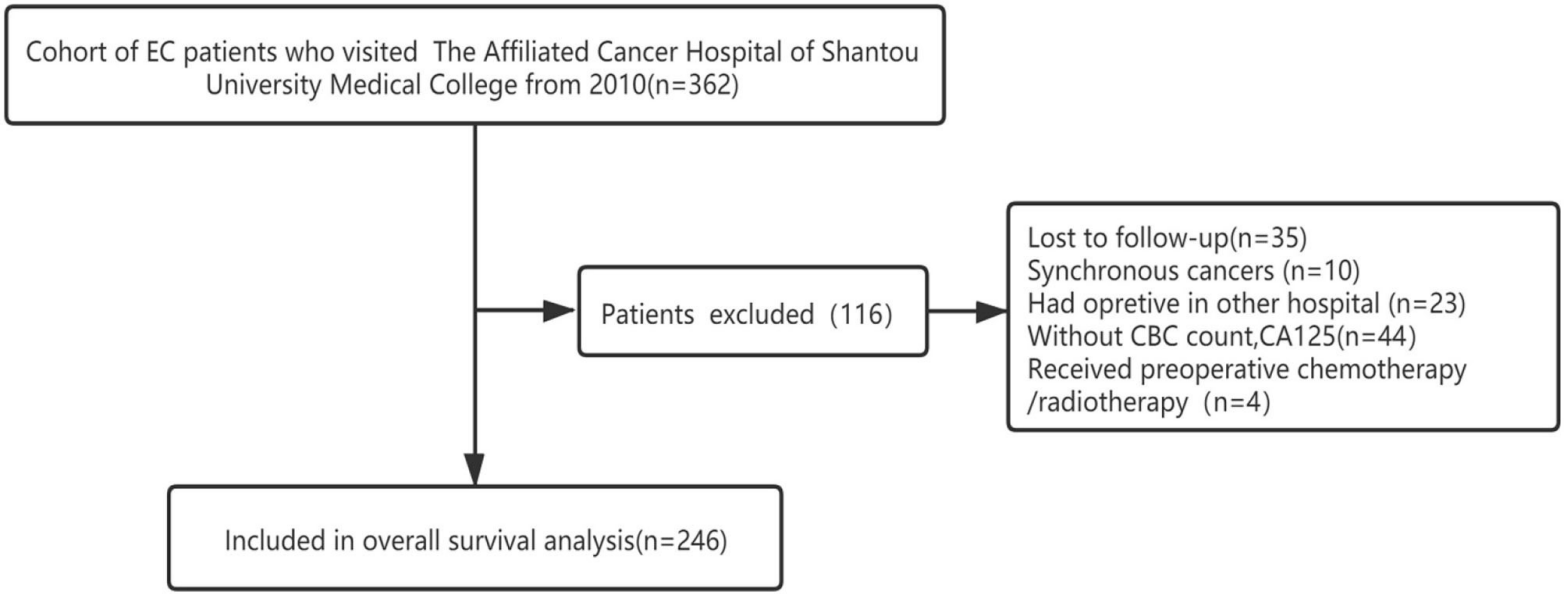
The flowchart of the enrollment process.

The flow chart of the screening progress is clearly shown in Figure 1; 116 patients were excluded in all, including 10 patients who were found to have synchronous cancers, 44 patients who lack medical data, 23 patients who received surgery in other hospitals, four patients who got adjuvant therapy before surgery, and 35 patients who were lost to follow-up. In the end, 246 patients were enrolled in our research.

Patient baseline characteristics are listed in Table 1. The mean age of patients is 54.17 ± 8.38 years old (range 25–75), and the mean follow-up time was 89.62 ± 1.22 months (range 23–125 months). At the end of the last follow-up, eight (3.3%) patients died. There are 135 (54.9%) patients with BMI ≤ 25 kg/m^2^, and 111 (45.1%) patients with BMI>25 kg/m^2^. In addition, 211 (85.5%) patients with stage I–II and 35 (14.2%) patients were diagnosed at stage III–IV. There were 65 (26.4%) and 144 (58.5%) patients that had tumor grade 1 and 2, respectively, and 11 (4.5%) patients were grade 3 while 25 (10.2%) patients were not possible to accurately tumor grade. There were 229 (93.1%) patients that were diagnosed with type I cancer and 17(6.9%) patients diagnosed with type II cancer. LVSI and deep myometrial invasion (MI, defined as ≥ 1/2 invasion) were present in 8 (3.3%) and 39 (15.9%) patients, respectively. Eighteen (7.3%) patients had positive regional lymph nodes. There were 142 (57.7%) patients who were in menopause status, and 50 (20.3%) and 196 (79.7%) patients had with the surgery approach of laparoscopy and laparotomy, respectively. Ninety-one (37%) patients had hypertension, and 40 (16.3%) had diabetes. Eighty-two (33.3%) patients had chemotherapy during the operative with cisplatin (DDP). Forty-seven (19.1%) and 28 (11.4%) patients had postoperative chemotherapy and postoperative radiotherapy.

**Table 1:** Baseline characteristics of the 246 patients.

**Variable**	**N(%)**	**Mean (SD)**
Age at surgery (year)		54.171 ± 8.38
≤ 54	114 (46.3)	
>54	132 (53.7)	
BMI		25.0466 ± 4.28
≤ 25	135 (54.9)	
>25	111 (45.1)	
**FIGO stage**		
Stage I-II	211 (85.8)	
Stage III-IV	35 (14.2)	
**Tumor grade**		
G1	65 (26.4)	
G2	144 (58.5)	
G3	11 (4.5)	
Unknown	25 (10.2)	
**Histology**		
Type I	229 (93.1)	
Type II	17 (6.9)	
**Lymphatic invasion**		
Positive	18 (7.3)	
Negative	228 (92.7)	
**LVSI**		
Positive	8 (3.3)	
Negative	238 (96.7)	
**Depth of myometrial invasion**		
<1/2	207 (84.1)	
≥1/2	39 (15.9)	
**Menopause**		
Yes	142 (57.7)	
No	104 (42.3)	
**Recurrence**		
Yes	10 (4.1)	
No	236 (95.9)	
**Death**		
Yes	8 (3.3)	
No	238 (96.7)	
**Surgical approach**		
Laparoscopy	50 (20.3)	
Laparotomy	196 (79.7)	
**Hypertension**		
Yes	91 (37)	
No	155 (63)	
**Diabetes**		
Yes	40 (16.3)	
No	206 (83.7)	
**Intraoperative chemotherapy(DDP)**		
Yes	82 (33.3)	
No	164 (66.7)	
**Postoperative chemotherapy**		
Yes	47 (19.1)	
No	199 (80.9)	
**Postoperative radiotherapy**		
Yes	28 (11.4)	
No	218 (88.6)	
Combine UM	76(30.9)	

*LVSI, lymphovascular space invasion; UM, myoma of the uterus; DDP, cisplatin*.

### The Optimal Cutoff Value of the Preoperative SII, NLR, PLR, MLR, CRP, CA125, and Postoperative SII

**Figure 2:**
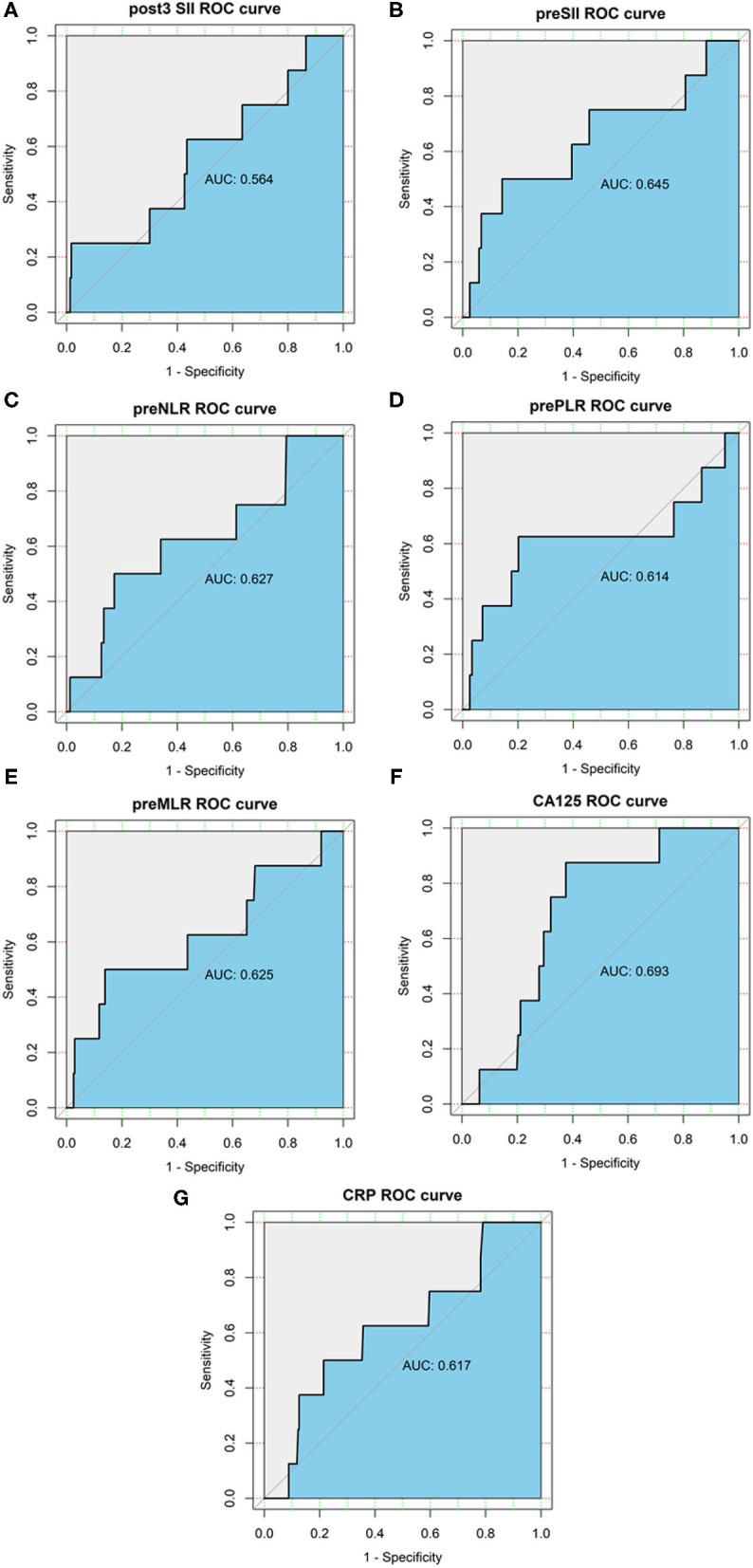
Receiver operating characteristics (ROC) curve analysis of the preoperative SII, NLR, PLR, MLR, CRP, CA125, and postoperative SII. **(A)** ROC curve analysis of the postoperative SII for OS in endometrial cancer patients. **(B)** ROC curve analysis of the preoperative SII for OS in endometrial cancer patients. **(C)** ROC curve analysis of the preoperative NLR for OS in endometrial cancer patients. **(D)** ROC curve analysis of the preoperative PLR for OS in endometrial cancer patients. **(E)** ROC curve analysis of the preoperative MLR for OS in endometrial cancer patients. **(F)** ROC curve analysis of the preoperative CA125 for OS in endometrial cancer patients. **(G)** ROC curve analysis of the preoperative CRP for OS in endometrial cancer patients.

As shown in Figure 2, the AUC was 0.645, 0.627, 0.614, 0.625, 0.617, 0.693, and 0.564 for the preoperative SII, NLR, PLR, MLR, CRP, CA125, and postoperative SII, respectively. The optimal cutoff value for the prediction of survival was 9.02 × 10^11^ for preoperative SII, 3.01 for preoperative NLR, 169.62 for preoperative PLR and 0.31 for preoperative MLR 3.73 for preoperative CRP, 24.58 for CA125, 2.93 × 10^12^ for postoperative SII. The patients were divided into high and low groups by the optimal cutoff value for further analysis. There were 38 (15.4%) patients who had the preoperative SII ≥ 9.02 × 10^11^, 208 (84.6%) patients had the preoperative SII <9.02 × 10^11^. There were 45 (18.3%) patients who had preoperative NLR ≥ 3.01, 201 (81.7%) patients who had preoperative NLR <3.01, 53 (21.5%) patients who had preoperative PLR ≥ 169.62, and 193 (78.5%) patients who had preoperative PLR < 169.62. Thirty-seven (15%) patients had preoperative MLR ≥ 0.31, 209 (85%) patients had preoperative MLR < 0.31, 6 (2.4%) patients had the postoperative SII ≥ 2.93 × 10^12^, 239 (97.6%) patients had the postoperative SII < 2.93 × 10^12^.

### Correlation Between the Clinical Variables and Preoperative SII, NLR, PLR, MLR, CRP, CA125, and Postoperative SII

The relationship between the clinical variables and preoperative SII, NLR, PLR, MLR, and postoperative SII is shown in Table 2. The preoperative SII was related to age (*p* = 009), FIGO stage (*p* = 0.02), and menopause (*p* = 0.014). The preoperative NLR was associated with age (*p* = 0.018), FIGO stage (*p* = 0.03), lymphatic invasion (*p* = 0.042) and menopause (*p* = 0.049). The preoperative PLR was associated with age (*p* < 0.001), FIGO stage (*p* = 0.004), tumor grade (*p* = 0.021), lymphatic invasion (*p* = 0.002), depth of myometrial invasion (*p* = 0.017), menopause (*p* < 0.001). The preoperative MLR was associated with tumor grade (*p* = 0.01), histology (*p* = 0.015), lymphatic invasion (*p* = 0.003), and menopause (*p* = 0.037). The postoperative SII was associated with menopause (*p* = 0.014).

**Table 2:** Correlations between the preoperative SII, NLR, PLR, MLR, postoperative SII, and clinicopathological variables in patients with endometrial cancer.

**Variable**	**Pre-SII**		***p-*value**	**Pre-NLR**		***p-*value**	**Pre-PLR**		***p*-value**	**Pre-MLR**		***p*-value**	**Post-SII**		***p-*value**
	≤	>		≤	>		≤	>		≤	>		≤	>	
Age at surgery (year)			0.009			0.018			<0.001			0.507			0.157
≤ 54	89 (78.1)	25 (21.9)		86 (42.80)	28 (62.20)		77 (39.90)	37 (69.80)		95 (45.50)	19 (51.40)		109 (95.6)	5 (4.4)	
>54	119 (90.2)	13 (9.8)		115 (57.20)	17 (37.80)		116 (60.10)	16 (30.20)		114 (54.50)	18 (48.60)		130 (99.2)	1 (0.8)	
BMI			0.265			0.154			0.065			0.334			0.312
≤ 25	111 (82.2)	24 (17.8)		106 (52.70)	29 (64.40)		100 (51.80)	35 (66.00)		112 (53.60)	23 (62.20)		129 (96.3)	5 (3.7)	
>25	97 (87.4)	14 (12.6)		95 (47.30)	16 (35.60)		93 (48.20)	18 (34.00)		97 (46.40)	14 (37.80)		110 (99.1)	1 (0.9)	
FIGO stage			0.02			0.03			0.004			0.056			0.448
Stage I-II	183 (86.7)	28 (13.3)		177 (88.10)	34 (75.60)		172 (89.10)	39 (73.60)		183 (87.60)	28 (75.70)		206 (98.1)	4 (1.9)	
Stage III-IV	25 (74.1)	10 (28.6)		24 (11.90)	11 (24.40)		21 (10.90)	14 (26.40)		26 (12.40)	9 (24.30)		33 (94.3)	2 (5.7)	
Tumor grade			0.093			0.267			0.021			0.01			0.767
G1-G2	178 (84.8)	32 (15.2)		174 (86.6)	36 (80)		169 (87.60)	41 (77.40)		183 (87.60)	27 (73.00)		204 (97.6)	5 (2.4)	
G3	7 (63.6)	4 (36.4)		7 (3.5)	4 (8.9)		5 (2.60)	6 (11.30)		6 (2.90)	5 (13.50)		11 (100)	0 (0)	
Unknown	23 (92)	2 (8)		20 (10)	5 (11.1)		19 (9.80)	6 (11.30)		20 (9.60)	5 (13.50)		24 (96)	1 (4)	
Histology			0.543			0.943			1.000			0.015			0.892
Type I	195 (85.2)	34 (14.8)		187 (93.00)	42 (93.30)		180 (93.30)	49 (92.50)		198 (94.70)	31 (83.80)		223 (97.8)	5 (2.2)	
Type II	13 (76.5)	4 (23.5)		14 (7.00%)	3 (6.70)		13 (6.70)	4 (7.50)		11 (5.30)	6 (16.20)		16 (94.1)	1 (5.9)	
Lymphatic invasion			0.065			0.042			0.002			0.003			0.093
Positive	12 (66.7)	6 (33.3)		11 (5.50)	7 (15.60)		184 (95.30)	44 (83.00)		11 (5.30)	7 (18.90)		16 (88.9)	2 (11.1)	
Negative	196 (86)	32 (14)		190 (94.5)	38 (84.4)		9 (4.70)	9 (17.00)		198 (94.70)	30 (81.10)		223 (98.2)	4 (1.8)	
LVSI			0.209			0.335			0.497			0.765			1.000
Positive	5 (62.5)	3 (37.5)		5 (2.5)	3 (6.7)		5 (2.60)	3 (5.70)		6 (2.90)	2 (5.40)		8 (100)	0 (0)	
Negative	203 (85.3)	35 (17.4)		196 (97.5)	42 (93.3)		188 (97.40)	50 (94.30)		203 (97.10)	35 (94.60)		231 (97.5)	6 (2.5)	
Depth of myometrial invasion			0.151			0.400			0.017			0.297			0.538
<1/2	178 (86)	29 (14)		171 (85.10)	36 (80.00)		168 (87.00)	39 (73.60)		178 (85.20)	29 (78.40)		202 (98.1)	4 (1.9)	
≥1/2	30 (76.9)	9 (23.1)		30 (14.90)	9 (20.00)		25 (13.00)	14 (26.40)		31 (14.80)	8 (21.60)		37 (94.9)	2 (5.1)	
menopause			0.014			0.049			<0.001			0.037			0.014
Yes	126 (89.4)	15 (10.6)		121 (60.50)	20 (44.40)		123 (64.10)	18 (34.00)		126 (60.30)	15 (41.70)		140 (100)	0 (0)	
No	81 (77.9)	23 (22.1)		79 (39.50)	25 (55.60)		69 (35.90)	35 (66.00)		83 (39.70)	21 (58.30)		98 (94.2)	6 (5.8)	
Combine UM			0.507			0.497			0.9			0.087			1.000
Yes	66 (86.8)	10 (13.2)		64 (31.80)	12 (26.70)		60 (31.10)	16 (30.20)		69 (33.00)	7 (18.90)		74 (97.4)	2 (2.6)	
No	142 (83.5)	28 (16.5)		137 (68.20)	33 (73.30)		133 (68.90)	37 (69.80)		140 (67.00)	30 (81.10)		165 (97.6)	4 (2.4)	
Surgical approach			0.751			0.952			0.93			0.264			1.000
Laparoscopy	43 (86)	7 (14)		41 (20.40)	9 (20.00)		39 (20.20)	11 (20.80)		45 (21.50)	5 (13.50)		49 (98)	1 (2)	
Laparotomy	165 (84.2)	31 (15.8)		160 (79.60)	36 (80.00)		154 (79.80)	42 (79.20)		164 (78.50)	32 (86.50)		190 (97.4)	5 (2.6)	
Hypertension			0.264			0.366			0.403			0.321			1.000
Yes	80 (87.9)	11 (12.1)		77 (38.30)	14 (31.10)		74 (38.30)	17 (32.10)		80 (38.30)	11 (29.70)		88 (97.8)	2 (2.2)	
No	128 (82.6)	27 (17.4)		124 (61.70)	31 (68.90)		119 (61.70)	36 (67.90)		129 (61.70)	26 (70.30)		151 (97.4)	4 (2.6)	
Diabetes			0.573			0.231			0.795			0.338			0.561
Yes	35 (87.5)	5 (12.5)		30 (14.90)	10 (22.20)		32 (16.60)	8 (15.10)		32 (15.30)	8 (21.60)		38 (95)	2 (5)	
No	173 (84)	33 (16)		171 (85.10)	35 (77.80)		161 (83.40)	45 (84.90)		177 (84.70)	29 (78.40)		201 (98)	4 (2)	
Intraoperative chemotherapy (DDP)			0.803			0.726			0.913			0.377			1.000
Yes	70 (85.4)	12 (14.6)		68 (33.80)	14 (31.10)		64 (33.20)	18 (34.00)		72 (34.40)	10 (27.00)		80 (97.6)	2 (2.4)	
No	138 (84.1)	26 (15.9)		133 (66.20)	31 (68.90)		129 (66.80)	35 (66.00)		137 (65.60)	27 (73.00)		159 (97.5)	4 (2.5)	
Postoperative chemotherapy			0.435			0.314			0.055			0.637			1.000
Yes	38 (80.9)	9 (19.1)		36 (17.90)	11 (24.40)		32 (16.60)	15 (28.30)		39 (18.70)	8 (21.60)		46 (97.9)	1 (2.1)	
No	170 (85.4)	29 (14.6)		165 (82.10)	34 (75.60)		161 (83.40)	38 (71.70)		170 (81.30)	29 (78.40)		193 (97.5)	5 (2.5)	
Postoperative radiotherapy			0.923			0.329			0.147			1.00			1.000
Yes	23 (82.1)	5 (17.9)		21 (10.40)	7 (15.60)		19 (9.80)	9 (17.00)		24 (11.50)	4 (10.80)		27 (96.4)	1 (3.6)	
No	185 (84.9)	33 (15.1)		180 (89.60)	38 (84.40)		174 (90.20)	44 (83.00)		185 (88.50)	33 (89.20)		212 (97.7)	5 (2.3)	

*LVSI, lymphovascular space invasion; UM, myoma of uterus; SII, systemic immune-inflammation index; NLR, neutrophil-to-lymphocyte ratio; PLR, platelet-to-lymphocyte ratio; MLR, monocyte-to-lymphocyte ratio; CA125, cancer antigen 125; pre-, preoperative; post3-, postoperative 3 days; DDP, cisplatin*.

*The yellow shade highlighted that P-value less than 0.05 was statistical significance*.

### Univariate and Multivariate Analyses

Univariate OS analyses demonstrated that FIGO stage (*P* < 0.001), lymphatic invasion (*P* < 0.001), depth of myometrial invasion (*P* = 0.002), postoperative chemotherapy (*P* = 0.007), postoperative radiotherapy (*P* < 0.001), and preoperative SII (*P* = 0.014), NLR (*P* = 0.029), PLR (*P* = 0.012), MLR (*P* = 0.01), CRP (*P* = 0.049), CA125 (*P* = 0.019), and postoperative SII (*P* < 0.001) were associated with OS. Age at surgery, BMI, tumor grade, histology, LVSI, menopause, whether combined with UM, surgical approach, intraoperative chemotherapy(DDP), and patients with diabetes or hypertension were not prognosis factors of OS (Table 3). Multivariate analysis with a stepwise procedure demonstrated that lymphatic invasion (HR: 21.35, 95% CI: 4.778–95.406, *p* < 0.0001) and postoperative SII (HR: 8.735, 95% CI: 1.447–51.646, *p* = 0.017) were significant independently predictive factors (Table 4).

**Table 3:** Univariate analysis of patient survival (*n* = 246).

**Variable**	**Overall survival**	
	**HR (95%CI)**	***P*-value**
Age at surgery (year, ≤ 54, >54)	6.163 (0.758–50.097)	0.089
BMI (≤ 25,>25)	1.152 (0.288–4.611)	0.841
FIGO stage (I-II/III-IV)	20.033 (4.04–99.343)	<0.001
Tumor grade (G1-G2/G3/unknown)	1.545 (0.673–3.547)	0.305
Histology (typeI, typeII)	4.355 (0.878–21.603)	0.072
Lymphatic invasion (positive/negative)	26.864(6.366–113.363)	<0.001
LVSI (positive/negative)	4.318 (0.531–35.126)	0.171
Depth of myometrial invasion (<1/2, ≥1/2)	10.067 (2.397–42.271)	0.002
Menopause (yes, no)	1.22 (0.292–5.107)	0.785
Combine UM (yes, no)	1.324 (0.316–5.54)	0.701
Surgical approach (laparoscopy, laparotomy)	1.170 (0.138–9.94)	0.885
Hypertension (yes, no)	0.556 (0.114–2.805)	0.486
Diabetes (yes, no)	1.771 (0.357–8.773)	0.484
Intraoperative chemotherapy(DDP)(yes, no)	0.520 (0.104–2.609)	0.427
Postoperative chemotherapy (yes, no)	7.222 (1.726–30.225)	0.007
Postoperative radiotherapy (yes, no)	13.655 (3.262–57.156)	<0.001
Pre-SII (>9.02 × 10^11^, ≤ 9.02 × 10^11^)	5.681 (1.421–22.719)	0.014
Pre-NLR (>3.01, ≤ 3.01)	4.704 (1.176–18.817)	0.029
Pre-PLR (>169.62, ≤ 169.62)	6.329 (1.512–26.488)	0.012
CA125 (>24.58, ≤ 24.58)	12.213 (1.501–99.395)	0.019
Pre-CRP (>3.73, ≤ 3.73)	4.053 (1.005–16.344)	0.049
Post3-SII (>2.93 × 10^12^, ≤ 2.93 × 10^12^)	19.589 (3.823–100.382)	<0.001
Pre-MLR (>0.31, ≤ 0.31)	6.23 (1.556–24.943)	0.01

*LVSI, lymphovascular space invasion; UM, myoma of uterus; SII, systemic immune-inflammation index; NLR, neutrophil-to-lymphocyte ratio; PLR, platelet-to-lymphocyte ratio; MLR, monocyte-to-lymphocyte ratio; CA125, cancer antigen 125; pre-, preoperative; post3-, postoperative 3 days; DDP, cisplatin*.

*The yellow shade highlighted that P-value less than 0.05 was statistical significance*.

**Table 4:** Multivariate analysis of patient survival (*n* = 246).

**Variable**	**Overall survival**	
	**HR (95%CI)**	***P*-value**
Lymphatic invasion (positive/negative)	21.35 (4.778−95.406)	<0.0001
Post3-SII (>2.93 × 10^12^, ≤ 2.93 × 10^12^)	8.735 (1.447–51.646)	0.017

*SII, systemic immune-inflammation index; post3-, postoperative 3 days*.

*The yellow shade highlighted that P-value less than 0.05 was statistical significance*.

### The Prognostic Value of Preoperative SII, NLR, PLR, MLR, CRP, CA125, and Postoperative SII

**Figure 3:**
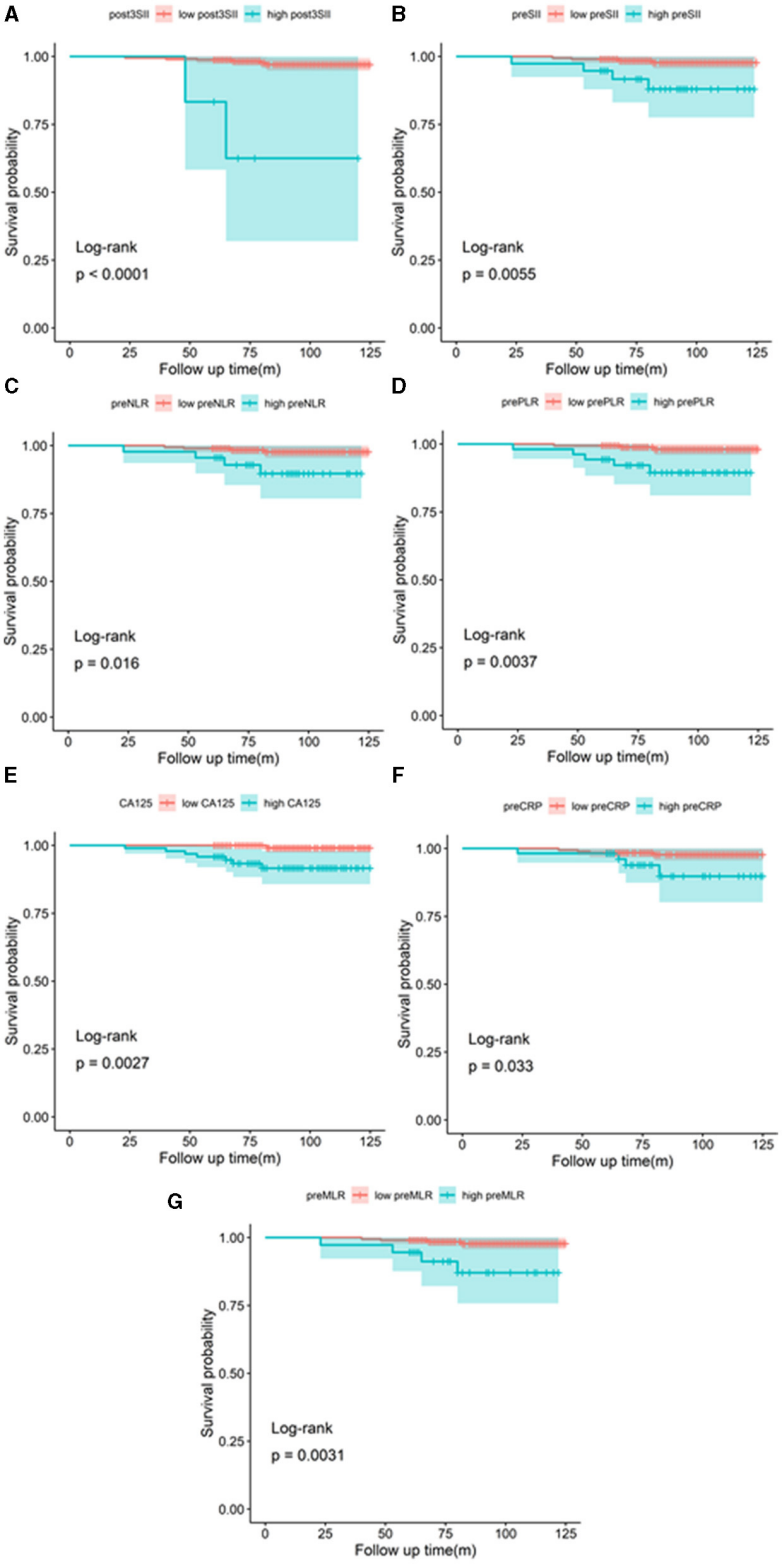
Kaplan–Meier curves of OS according to the postoperative SII **(A)**, preoperative SII **(B)**, NLR **(C)**, PLR **(D)**, CA125 **(E)**, CRP **(F)**, and MLR **(G)**.

**Figure 4:**
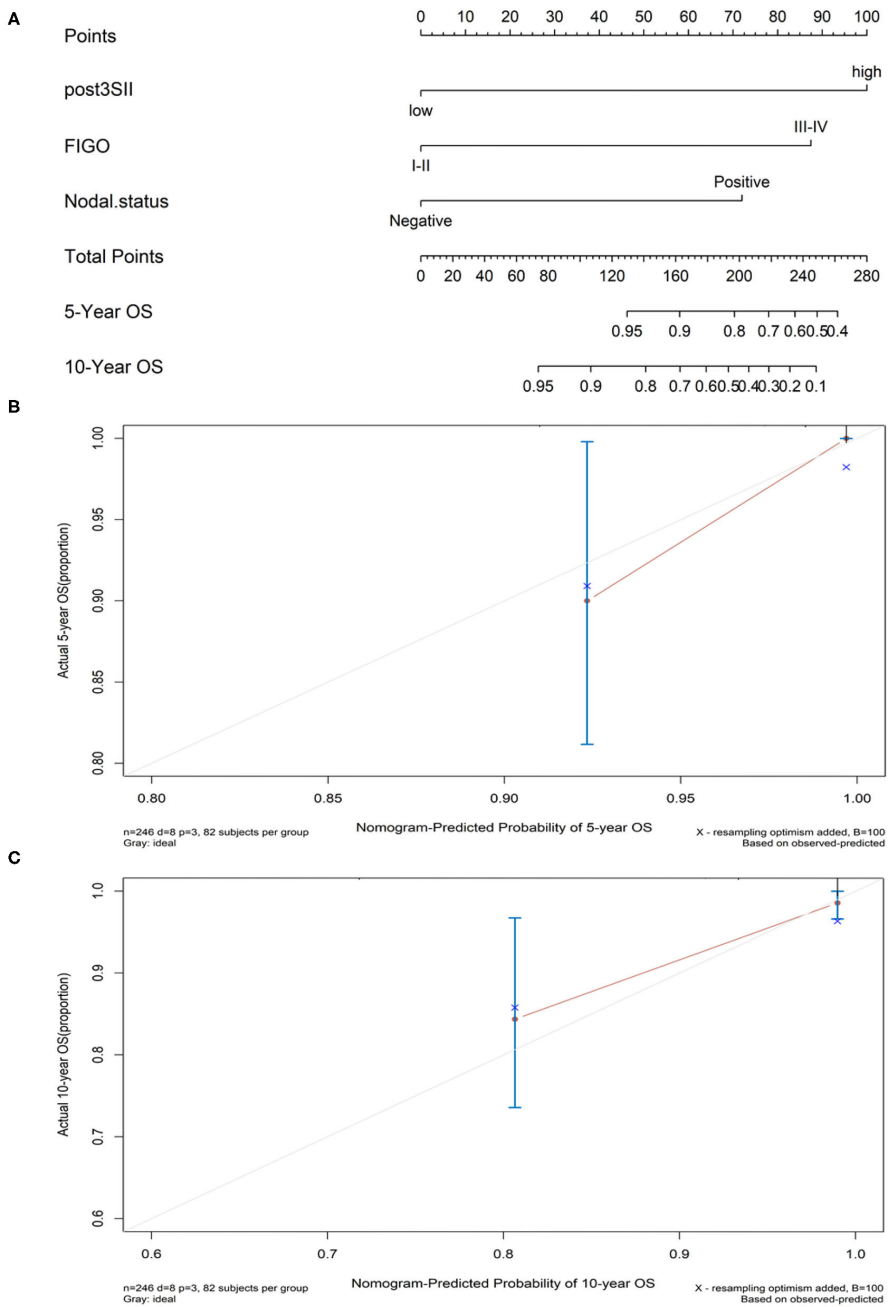
**(A)** Nomogram model for estimating the rate of OS (5 or 10 years) for women with endometrial cancer. **(B,C)** Calibration curves of the nomograms. The calibration curves predicted OS at 5 years **(B)** and 10 years **(C)**. Nomogram-predicted probability of OS was plotted on the *x*-axis and the observed OS was plotted on the *y*-axis.

The Kaplan–Meier method with the log-rank test was used to generate the OS curves. High preoperative SII, NLR, PLR, MLR, CRP, and CA125 and postoperative SII were associated with poor OS (*p* = 0.0055, *p* = 0.016, *p* = 0.0037, *p* = 0.0031, *p* = 0.033, *p* = 0.0027, and *p* < 0.0001, respectively) (Figure 3). A nomogram model was formed according to multivariate Cox regression analysis with composite indicators (postoperative SII, FIGO, nodal status) to visualize and assess the prognostic value of OS in endometrial cancer operation (Figure 4). The Harrell's C-index of the nomogram was 0.866. The C-index of the nomogram model without the involvement of FIGO declined to 0.812. The calibration curves indicated good consistency of the nomogram-predicted probability of 5- and 10-year OS (Figure 4).

## Discussion

Systemic inflammation plays an important role in the occurrence and development of cancer. Inflammatory biomarkers are conformed to the outward manifestation of the response of antitumor immunity. This study first investigated the relationship between postoperative SII and the prognosis of patients diagnosed with endometrial cancer after operative treatment. Our results indicated that the postoperative SII predicted the prognostic value for endometrial carcinoma patients undergoing surgery.

As we have seen, there has been no consistent opinion about the perfect time to get a complete blood count to figure out the inflammation index after the operation. Some studies limit the proper time of postoperative inflammatory biomarkers to within 1 week after the procedure (16–18). Meanwhile, some literature considered that the operative trauma causing systemic inflammatory reactions might cease until 1 month later (19). The inflammatory condition can be changed after the removal of the tumor. Postoperative systemic immune inflammation could precisely reflect the internal inflammatory state of patients getting rid of the tumor-carrying status. The postoperative inflammation was supposed to provide effective guidance about follow-up treatment after surgery. The persistent occurrance of postoperative systemic inflammation may cause progress of the tumor (20). Tissue injury and inflammatory response after surgery are considered to be important factors affecting postoperative recovery. The study shows that laparoscopic surgery is associated with a lower incidence of postoperative complications and a short-term improvement in quality of life compared to open surgery (21). Laparoscopic hysterectomy also has lower activation of the inflammatory response, less impact on cellular immunity, and different inflammatory responses. However, in this research, two surgical approaches were not associated with systemic immune inflammation indicators nor were they significant with the survival prognosis of patients with endometrial cancer. The infection may influence the systemic immune inflammation factors after surgery and chronic inflammation. However, patients with chronic inflammation were excluded from our study group, and the infection after operation often happened 5–7 days after surgery. In this research, an aliquot of blood was taken the third day after surgery to exclude the influence of wound infection.

Inflammation plays a vital part in tumor initiation, metastasis and the host antitumor immunity. Neutrophils contribute to the mechanism of several diseases. Neutrophil counts may increase following operations and then transform the tumor microenvironment of the host due to the recurrence and metastasis (22). Granulocyte-colony stimulating factor (GCSF) may gain a high level by the stimulation of cancer cells, which activate the signaling pathway of Janus Kinase (JAK)-signal transducer and activator of transcription 3(STAT3), contributing to the migration and proliferation of neutrophil (23). A finding shows that neutrophils escort circulating tumor cells, increasing tumor metastasis risk (24). Circulating tumor cells collectively refer to several kinds of tumor cells present in peripheral blood (25). Matrix metalloproteinase (MMPs) and vascular endothelial growth factor (VEGF) released by neutrophils could induce tumor angiogenesis, promoting the growth and metastasis of the tumor (26). Neutrophils suppressing antitumor immunity may be caused by the exaggerated inflammatory (27).

Platelets are proved to have interaction with tumor cells. Tumor cells could activate platelets by secreting thrombin and expressing tissue factors, which form a physical barrier of a platelet and fibrous protein grid in which cancer cells could be hidden escaping the surveillance of natural killer cells (NK cells) (28). Angiogenesis provides oxygen and nutrition and removes the toxic substance produced in the tumor microenvironment, facilitating tumor growth (23, 29, 30). Reduced numbers of lymphocytes are often predictive of tumor recurrence. Previous research shows that nuclear factor-κB (NF-κB) and inflammation protect the circulating tumor cells against death due to the epithelial–mesenchymal transition (EMT) (31).

The combination of neutrophils, platelets, and lymphocytes is most comprehensive to reveal the relationship between cancer cells and systematic immune-inflammatory. On the third day after the operation, high neutrophil and platelet counts and low counts of lymphocytes led to the high postoperative SII, indicating an exaggerated inflammatory and a let-up immune protection. Therefore, the higher postoperative SII is closely related to tumor progression and poor outcomes of endometrial cancer patients after surgery. It is valuable to make a prospective and comprehensive clinical protocol according to the postoperative SII for patients diagnosed with endometrial cancer.

Neutrophil-lymphocyte ratio (NLR), platelet-lymphocyte ratio (PLR), and monocyte-lymphocyte ratio (MLR) are associated with poor OS in previously published reports (14, 32). However, preoperative NLR, PLR, MLR, CA 125, and CRP were not independent prognostic factors in our study. The SII comprises lymphocyte, neutrophil, and platelet counts, which more precisely predict prognosis than PLR, NLR, and MLR and reflect the actual status of tumor-associated immunoreaction just like the previous study showed that higher FIGO stage, lymph node invasion, and deeper depth of myometrial was supposed to be significantly correlated with poor prognosis (7, 33).

On the contrary, the clinical recognized risk factors, such as obesity, hypertension, and diabetes did not significantly affect prognosis in our study. Some findings showed that metformin in patients with endometrial cancer has a good OS (34–36). However, the recording of using metformin in our study was incomplete so that whether this may affect endometrial cancer patients' prognosis or not is unclear and needs in-depth research. Postoperative radiotherapy and chemotherapy are usually used in patients with advanced stage or dissatisfied operation in the clinic to reduce the risk of recurrence or metastases, which may have collinearity bias in Cox regression analysis (37).

A nomogram is the visualization of the regression model, which evaluates the prognosis in clinical practice. To our knowledge, the clinical nomogram model combines several independent risk factors to predict the prognosis of every unique patient and considers the value of subsequent chemotherapy or radiotherapy after surgery, which can ably help clinicians making accurate and effective decisions (38, 39). This research built the clinical nomogram model consisting of postoperative SII, FIGO, and nodal status based on multiple regression analysis. The reason we add the variable of the FIGO stage was to consider the broad applicability in the clinic. The guideline of the clinical nomogram model indicates that the concordance index (C-index) may have a precisely predictive value if the number is closing to one (39). The C-index of this clinical nomogram model is 0.866, which could exactly predict the OS of endometrial cancer patients. The calibration curves show good discrimination and calibration of the nomogram of 5- and 10-year OS.

One of the limitations in this study is that the specimen adequacy is not satisfied, and the character of review analysis may generate selection bias. Another is the design of single-center analysis. Hence, it is important to design a study with a large sample, prospective and multicenter, that prove the results of our study.

## Conclusion

This is the first study to assess the predicting prognostic value of preoperative and postoperative SII in endometrial cancer patients after surgery. Postoperative SII, rather than preoperative SII, is independent prognostic factors for OS in endometrial cancer patients after the standard operation. In clinical practice, patients could be divided into high and low groups according to the postoperative SII and make good use of the nomogram model to make an individualized assessment for better subsequent therapy.

## Data Availability Statement

The original contributions presented in the study are included in the article/supplementary material, further inquiries can be directed to the corresponding authors.

## Ethics Statement

The studies involving human participants were reviewed and approved by Cancer Hospital of Shantou University Medical College. The patients/participants provided their written informed consent to participate in this study. Written informed consent was obtained from the individual(s) for the publication of any potentially identifiable images or data included in this article.

## Author Contributions

YH, YC, YZ, and CL acquired data, analyzed, and interpreted data and statistical analysis. YH and HX drafted the manuscript and critically revised the manuscript content. YC, YZ, and QW provided clinical or material support. CY contributes some sample analysis and funding support. All authors approved the final version of the manuscript.

## Funding

This study was supported by grants from the Recruitment Program of Overseas High-Level Young Talents, Innovative and Entrepreneurial Team [No. (2018) 2015] of Jiangsu Province; Shantou grant numbers: [(2018) 121 (XQL)], Science and Technology Special Fund of Guangdong Province of China (190829105556145) and Strategic and Special Fund for Science and Technology Innovation of Guangdong Province of China (180918114960704); Head Goose Talent Funding of the Wuxi Maternal and Child Health Hospital Affiliated Nanjing Medical University; Beijing Medical and Health Public Welfare Foundation and Beijing Xisike Clinical Oncology Research Foundation (Y-HS202102-0177).

## Conflict of Interest

The authors declare that the research was conducted in the absence of any commercial or financial relationships that could be construed as a potential conflict of interest.

## Publisher's Note

All claims expressed in this article are solely those of the authors and do not necessarily represent those of their affiliated organizations, or those of the publisher, the editors and the reviewers. Any product that may be evaluated in this article, or claim that may be made by its manufacturer, is not guaranteed or endorsed by the publisher.
